# Healthcare seeking behavior for respiratory illness in a northern province of Vietnam

**DOI:** 10.1186/2047-2994-4-S1-P16

**Published:** 2015-06-16

**Authors:** YTT Nguyen, TB Nguyen, TP Nguyen, TH Nguyen, HH Vu, TV Nguyen, TH Pham, TT Do, HT Duong, LH Nguyen, JM Partridge, JC Kile, A Iuliano, HT Nguyen

**Affiliations:** 1 National Institute of Hygiene and Epidemiology, Hanoi, Viet Nam; 2 Provincial Preventive Medicine Center, Thai Binh, Viet Nam; 3 Program, U.S. Centers for Disease Control and Prevention, Hanoi, Viet Nam; 4 Bill and Melinda Gates Foundation, Seattle, USA; 5 Influenza Division, U.S. Centers for Disease Control and Prevention, Atlanta, USA

## Introduction

The national sentinel surveillance (NSS) system in Vietnam captures only cases presenting to sentinel sites, limiting our understanding of burden of disease in the community

## Objectives

To identify self-reported cases of influenza-like illness (ILI) and severe acute respiratory infection (SARI) in the community and describe healthcare utilization to better estimate the burden of influenza-associated illness

## Methods

A cross-sectional survey was conducted in Thai Binh Province. A two-stage cluster sample was used to select households. Standardized questionnaires were used to screen households for episodes of self-reported ILI in the previous month and SARI in the previous 12 months and health seeking behavior for each episode

## Results

We surveyed 2,100 households and 6,760 residents in May 2013, including 1,470 households and 4,666 residents in rural Kien Xuong District and 630 urban households and 2,094 residents in Thai Binh City. Overall, we identified 582 (9%, 95% CI: 6-11) episodes of self-reported ILI and 121 (2%, 95% CI: 1-2) episodes of self-reported SARI cases. The proportions of both self-reported ILI and SARI were significantly (p< 0.05) lower in Thai Binh City than in Kien Xuong. The proportion of cases of seeking healthcare outside the home for an ILI episode within the last month was 89% (95% CI: 84-94). Only 18% (95% CI: 10-27) of household members with a self-reported ILI episode sought healthcare at the ILI NSS site. The estimated proportion of SARI cases that sought healthcare within the last year at a SARI burden study site was 25%.

## Conclusion

In Thai Binh Province the majority of cases with self-reported ILI sought healthcare outside the home. However, less than 1/5 of self-reported ILI cases came to a national ILI NSS site. Similarly, only 1/4 of SARI cases treated at a SARI burden study site. The studies depend on healthcare seeking behavior of the populations that will underestimate burden of influenza-associated disease in Vietnam. Adjustment for healthcare utilization practices will accurately estimate the incidence of influenza in the community

## Disclosure of interest

None declared.

